# Clinical value of SUVpeak-to-tumor centroid distance on FDG PET/CT for predicting neoadjuvant chemotherapy response in patients with breast cancer

**DOI:** 10.1186/s40644-024-00787-4

**Published:** 2024-10-11

**Authors:** Sun-pyo Hong, Sang Mi Lee, Ik Dong Yoo, Jong Eun Lee, Sun Wook Han, Sung Yong Kim, Jeong Won Lee

**Affiliations:** 1 Department of Nuclear Medicine, Soonchunhyang University Cheonan Hospital, 31 Suncheonhyang 6-gil, Dongnam-gu, Cheonan, 31151 Republic of Korea; 2 Department of Surgery, Soonchunhyang University Cheonan Hospital, Cheonan, Republic of Korea

**Keywords:** Breast cancer, F-18 fluorodeoxyglucose, Neoadjuvant chemotherapy, Positron emission tomography, Radiomics

## Abstract

**Background:**

Since it has been found that the maximum metabolic activity of a cancer lesion shifts toward the lesion edge during cancer progression, normalized distances from the hot spot of radiotracer uptake to tumor centroid (NHOC) and tumor perimeter (NHOP) have been suggested as novel F-18 fluorodeoxyglucose (FDG) positron emission tomography/computed tomography (PET/CT) parameters that can reflect cancer aggressiveness. This study aimed to investigate whether NHOC and NHOP parameters could predict pathological response to neoadjuvant chemotherapy (NAC) and progression-free survival (PFS) in breast cancer patients.

**Methods:**

This study retrospectively enrolled 135 female patients with breast cancer who underwent pretreatment FDG PET/CT and received NAC and subsequent surgical resection. From PET/CT images, normalized distances of maximum SUV and peak SUV-to-tumor centroid (NHOCmax and NHOCpeak) and -to-tumor perimeter (NHOPmax and NHOPpeak) were measured, in addition to conventional PET/CT parameters.

**Results:**

Of 135 patients, 32 (23.7%) achieved pathological complete response (pCR), and 34 (25.2%) had events during follow-up. In the receiver operating characteristic (ROC) curve analysis, NHOCmax showed the highest area under the ROC curve value (0.710) for predicting pCR, followed by NHOCpeak (0.694). In the multivariate logistic regression analysis, NHOCmax, NHOCpeak, and NHOPmax were independent predictors for pCR (*p* < 0.05). In the multivariate survival analysis, NHOCpeak (*p* = 0.026) was an independent predictor for PFS along with metabolic tumor volume, with patients having higher NHOCpeak showing worse PFS.

**Conclusion:**

NHOCpeak on pretreatment FDG PET/CT could be a potential imaging parameter for predicting NAC response and survival in patients with breast cancer.

## Introduction

Breast cancer is the most commonly diagnosed cancer and, also, the leading cause of cancer death in women, exhibiting 2.3 million newly diagnosed cases with over 660,000 deaths globally in 2022 [1]. In patients with locally advanced breast cancer, neoadjuvant chemotherapy (NAC) followed by surgery has been the standard treatment, especially in those with human epidermal growth factor receptor 2 (HER2)-enriched or triple negative cancer types with a tumor size > 2 cm and/or axillary lymph node metastasis [2]. NAC has been shown to downstage breast cancer, increase the chance of reducing the extent of surgery, and achieve an overall survival comparable to conventional adjuvant chemotherapy [3, 4]. In breast cancer patients receiving NAC, pathological complete response (pCR) to NAC is known to be a major prognostic factor, consistently showing significant association with better survival outcomes [5, 6]. Therefore, many studies have tried to identify a predictive factor for pCR to allow optimal selection of treatment options and avoid toxicities of unnecessary treatment [7, 8].

In locally advanced breast cancer, F-18 fluorodeoxyglucose (FDG) positron emission tomography/computed tomography (PET/CT) has shown to be an effective imaging tool for pretreatment work-up by identifying significantly more distant metastatic lesions than conventional imaging examinations [9, 10]. Although previous studies have also investigated the clinical significance of pretreatment FDG PET/CT for predicting pCR in breast cancer patients treated with NAC, they have shown inconsistent results regarding the predictive value of conventional FDG PET/CT parameters such as maximum standardized uptake value (SUVmax), metabolic tumor volume (MTV), and total lesion glycolysis (TLG) [7, 8, 11, 12]. A recent study has mathematically modeled tumor growth and found that maximum metabolic activity moves toward the tumor edge during cancer progression [13]. Therefore, as a malignant tumor evolves with time, normalized distance from the hot spot of radiotracer uptake to the tumor centroid (NHOC) is increased, while normalized distance from the hot spot of radiotracer uptake to the tumor perimeter (NHOP) is decreased [13, 14]. These novel geometrical PET/CT parameters were considered to reflect tumor aggressiveness and associated with prognosis in patients with lung cancer and breast cancer [13, 14]. However, the prognostic significance of NHOC and NHOP parameters for predicting NAC response in patients with breast cancer has not been reported yet.

Thus, the goal of the present study was to investigate the clinical value of NHOC and NHOP parameters measured from pretreatment FDG PET/CT for predicting response to NAC and progression-free survival (PFS) in patients with breast cancer.

## Methods

### Patients

Medical records of female patients with histopathologically proven invasive breast carcinoma in two medical centers between January 2013 and December 2021 were retrospectively reviewed. Among these patients, 135 patients who underwent pretreatment FDG PET/CT and received NAC and subsequent surgical resection were selected in this study. Exclusion criteria were: (1) history of another malignant disease, (2) distant metastasis on pretreatment examinations, (3) no surgery after NAC, (4) small tumor volume (< 1 cm^3^) for calculating peak SUV (SUVpeak), (5) low FDG uptake in cancer tissue for delineating from normal breast tissue, and (6) follow-up loss within two years after the surgery without event.

For pretreatment assessment, blood tests, breast ultrasonography, contrast-enhanced CT, breast magnetic resonance imaging, bone scintigraphy, and FDG PET/CT were performed. Patients were treated with five different NAC regimens. Following NAC, mastectomy or breast conserving surgery with axillary lymph node dissection was conducted. pCR was defined as the absence of both invasive cancer cells and cancer in situ cells in breast and axillary lymph nodes (ypT0N0). Patients with pCR were considered complete responders, and patients who showed the presence of any residual invasive cancer cells were considered non-responders. For non-responders, the percentage of the primary tumor area composed of residual invasive cancer cells was measured and defined as residual tumor cellularity. After surgery, all enrolled patients received adjuvant chemotherapy, radiotherapy, and/or hormonal treatment.

### FDG PET/CT

Patients were fasted for at least 6 h before PET/CT scan with their blood glucose levels controlled below 150 mg/dL. Approximately 60 min after intravenous injection of 4.07 MBq/kg of FDG, PET/CT scanning was performed from the skull base to the proximal thigh using two dedicated PET/CT scanners (Biograph mCT 20 scanner and Biograph mCT 128 scanner, Siemens Healthineers, Knoxville, TN, USA). An unenhanced CT scan was performed at 80 mA and 100 kVp for the Biograph mCT 20 scanner and at 100 mA and 120 kVp for the Biograph mCT 128 scanner with a slice thickness of 5 mm. A PET scan was performed for 1.5 min per bed position with both scanners. Attenuation-correction PET images were reconstructed using the ordered-subset expectation maximization algorithm with the point spread function and time-of-flight modeling on a 128 × 128 matrix (2 iterations and 21 subsets).

### Image analysis

**Fig. 1:**
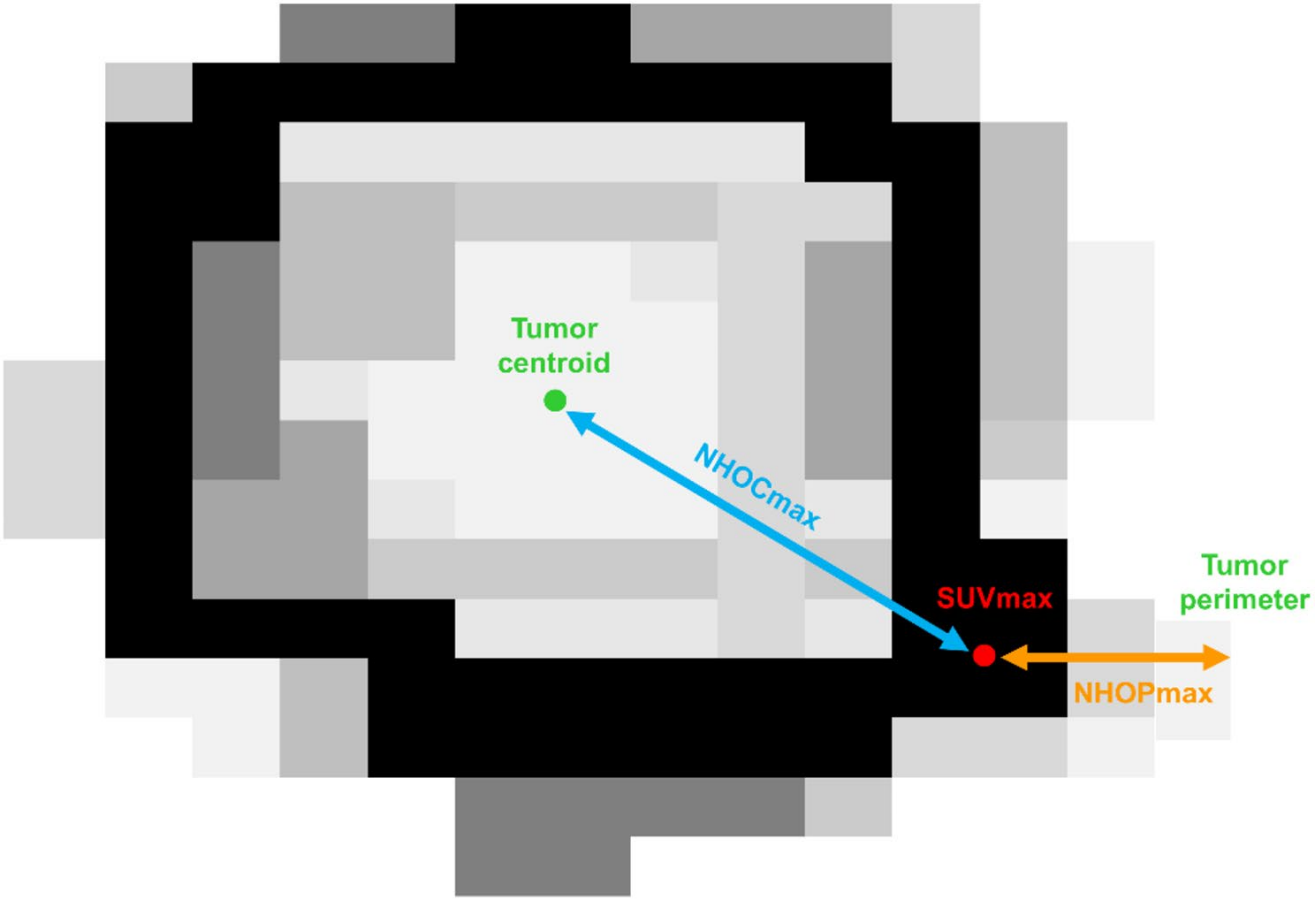
A schematic image regarding the measurement of NHOCmax and NHOPmax

Two experienced nuclear medicine physicians retrospectively analyzed FDG PET/CT images based on the consensus using the open-source LIFEx software version 7.6.0 (www.lifexsoft.org) compliant with the Image Biomarker Standardisation Initiative [15]. Prior to image analysis, all PET images were reconstructed into a voxel size of 4.07 × 4.07 × 2.5 mm. The metabolically active primary breast cancer lesion was automatically delineated using Nestle’s adaptive threshold method used in previous studies: (SUV threshold of the primary tumor) = 0.3 × (mean SUV of tumor voxels showing FDG uptake of > 70% of maximum SUV) + (mean SUV of background) [16, 17]. From the metabolically active primary cancer lesion, four conventional PET features (SUVmax, SUVpeak, MTV, and TLG) were calculated. Additionally, NHOC and NHOP parameters were measured based on a previously published method (Fig. 1) [14]. Distances from SUVmax voxel and center of voxels used for SUVpeak calculation to the tumor centroid divided by the radius of a hypothetical sphere of the tumor lesion were defined as NHOCmax and NHOCpeak, respectively. Likewise, distances from SUVmax voxel and center of voxels used for SUVpeak calculation to the tumor perimeter divided by the radius of the hypothetical sphere were defined as NHOPmax and NHOPpeak, respectively.

### Statistical analysis

The Mann–Whitney U test and Kruskal–Wallis test were performed to assess differences of FDG PET/CT parameters according to clinico-pathological factors. Spearman’s correlation coefficients were calculated to evaluate relationships of FDG PET/CT parameters with Ki-67 expression level and residual tumor cellularity. Abilities of FDG PET/CT parameters for predicting pCR were evaluated by calculating area under the receiver operating characteristic (ROC) curve (AUC) values. Using optimal cut-off values determined by the Youden index, sensitivity and specificity of PET/CT parameters for predicting pCR were identified. Univariate and multivariate logistic regression analyses were performed to investigate predictive values of PET/CT parameters for pCR. The prognostic significance of PET/CT parameters in predicting PFS was assessed using univariate and multivariate Cox proportional hazard regression analyses. PFS was defined as the duration from the day of NAC initiation to the day of disease progression detection, death, or last clinical follow-up. In the multivariate logistic regression and survival analyses, PET/CT parameters that showed statistical significance in the univariate analysis were included. Taking into account numbers of patients with events, PET/CT parameters were separately included in a multivariate model with age, T stage, N stage, and molecular subtypes added as covariates. Using the cut-off value determined by ROC curve analysis for predicting pCR, the Kaplan–Meier analysis was conducted for PFS curve estimation. PFS curves were compared between groups by the log-rank test. All statistical analyses were conducted using MedCalc Statistical Software version 22.021 (MedCalc Software Ltd, Ostend, Belgium). Statistical significance was set at *p* < 0.05.

## Results

### Patients characteristics

Characteristics of enrolled patients are summarized in Table 1. Of the 135 patients, 130 (96.3%) were diagnosed with invasive ductal carcinoma, and 125 (92.6%) were found to have lymph node metastases on pretreatment examinations. On pathological evaluation of surgical specimens, 32 patients (23.7%) and 103 patients (76.3%) were classified as complete responders and non-responders, respectively. The median value of residual tumor cellularity of 103 non-responders was 10% (range, 5–100%). The median duration of clinical follow-up was 60.0 months (range, 5.6–137.3 months), and, during follow-up, 34 patients (25.2%) had events.

**Table 1:** Characteristics of the enrolled patients (*n* = 135)

Characteristics		Number of patients (%)
Median age (range)		47 years (27–79 years)
Menopausal status	Premenopausal	75 (55.6%)
	Postmenopausal	60 (44.4%)
Histology	Invasive ductal carcinoma	130 (96.3%)
	Others	5 (3.7%)
Histologic grade	Grade 1	12 (8.9%)
	Grade 2	75 (55.6%)
	Grade 3	45 (33.3%)
	Not specified	3 (2.2%)
Molecular subtypes	Luminal A	27 (20.0%)
	Luminal B	71 (52.6%)
	HER2-enriched	19 (14.1%)
	Triple negative	18 (13.3%)
Median Ki-67 expression level (range)		30% (1–99%)
Clinical T stage	T1–T2	81 (60.0%)
	T3–T4	54 (40.0%)
Clinical N stage	N0	10 (7.4%)
	N1–3	125 (92.6%)
NAC regimen	Doxorubicin and docetaxel	50 (37.0%)
	Doxorubicin, cyclophosphamide, and docetaxel	35 (25.9%)
	Docetaxel, carboplatin, trastuzumab, and pertuzumab	24 (17.8%)
	Doxorubicin and cyclophosphamide	18 (13.3%)
	Doxorubicin, cyclophosphamide, paclitaxel, and trastuzumab	8 (5.9%)

### Correlation analysis of NHOC and NHOP parameters with clinico-pathological factors

Relationship of NHOC and NHOP parameters with clinico-pathological factors are shown in Table 2. Although there were no significant differences in values of NHOC and NHOP parameters according to molecular subtypes, patients with triple negative cancer showed significantly higher values of NHOCmax and NHOCpeak than others (*p* < 0.05). Patients with T3–T4 stage revealed significantly higher values of NHOCmax and NHOCpeak and lower value of NHOPpeak than those with T1–T2 stage (*p* < 0.05). On the contrary, clinical N stage showed no significant correlation with NHOC and NHOP parameters. Complete responders showed significantly higher values of NHOCmax and NHOCpeak but lower values of NHOPmax than non-responders.

**Table 2:** Correlation analysis of NHOC and NHOP parameters with clinico-pathological factors

Factors	NHOCmax	NHOCpeak	NHOPmax	NHOPpeak
Histology				
Invasive ductal carcinoma	0.56 (0.38–0.74)	0.43 (0.29–0.67)	0.22 (0.15–0.36)	0.28 (0.18–0.37)
Others	0.62 (0.47–0.86)	0.53 (0.31–0.83)	0.13 (0.11–0.35)	0.15 (0.12–0.42)
P-value	0.545	0.499	0.316	0.408
Histologic grade				
Grade 1	0.51 (0.40–0.90)	0.41 (0.34–0.75)	0.22 (0.11–0.35)	0.32 (0.20–0.42)
Grade 2	0.56 (0.37–0.79)	0.41 (0.27–0.70)	0.24 (0.16–0.37)	0.28 (0.19–0.37)
Grade 3	0.56 (0.39–0.72)	0.47 (0.31–0.57)	0.20 (0.14–0.35)	0.24 (0.15–0.38)
P-value	0.657	0.876	0.707	0.613
Molecular subtypes				
Luminal A	0.56 (0.38–0.70)	0.39 (0.27–0.63)	0.24 (0.15–0.37)	0.32 (0.16–0.37)
Luminal B	0.50 (0.36–0.74)	0.45 (0.25–0.68)	0.23 (0.15–0.36)	0.32 (0.16–0.36)
HER2-enriched	0.62 (0.39–0.70)	0.41 (0.30–0.54)	0.20 (0.12–0.36)	0.37 (0.19–0.48)
Triple negative	0.67 (0.48–0.87)	0.54 (0.39–0.81)	0.22 (0.19–0.30)	0.30 (0.19–0.35)
P-value	0.263	0.194	0.831	0.560
Triple negative cancer				
Triple negative	0.67 (0.48–0.87)	0.54 (0.39–0.81)	0.22 (0.19–0.30)	0.28 (0.16–0.38)
Others	0.54 (0.37–0.74)	0.41 (0.27–0.65)	0.22 (0.14–0.37)	0.30 (0.19–0.37)
P-value	0.049	0.033	0.885	0.698
Clinical T stage				
T1–T2	0.54 (0.32–0.71)	0.37 (0.27–0.58)	0.25 (0.17–0.36)	0.31 (0.19–0.39)
T3–T4	0.60 (0.42–0.87)	0.55 (0.38–0.72)	0.19 (0.12–0.36)	0.22 (0.15–0.35)
P-value	0.041	0.005	0.075	0.035
Clinical N stage				
N0	0.63 (0.44–0.78)	0.46 (0.30–0.63)	0.24 (0.19–0.37)	0.35 (0.28–0.46)
N1–N3	0.56 (0.37–0.74)	0.43 (0.29–0.67)	0.22 (0.15–0.35)	0.26 (0.17–0.37)
P-value	0.294	0.873	0.715	0.257
Pathological response				
Complete responder	0.43 (0.26–0.60)	0.30 (0.20–0.50)	0.33 (0.18–0.39)	0.32 (0.19–0.39)
Non-responder	0.59 (0.42–0.78)	0.47 (0.31–0.70)	0.21 (0.14–0.33)	0.25 (0.16–0.35)
P-value	< 0.001	0.009	0.028	0.114

Data are presented as median (interquartile range)

On correlation analysis, Ki-67 expression level showed no significant correlation with NHOC and NHOP parameters (*p* > 0.05). Meanwhile, residual tumor cellularity had significant positive, but weak, correlations with both NHOCmax (*r* = 0.264, *p* = 0.002) and NHOCpeak (*r* = 0.267, *p* = 0.002), but not with NHOPmax and NHOPpeak (*p* > 0.05).

### Correlation analysis of NHOC and NHOP parameters with pathological response

**Fig. 2:**
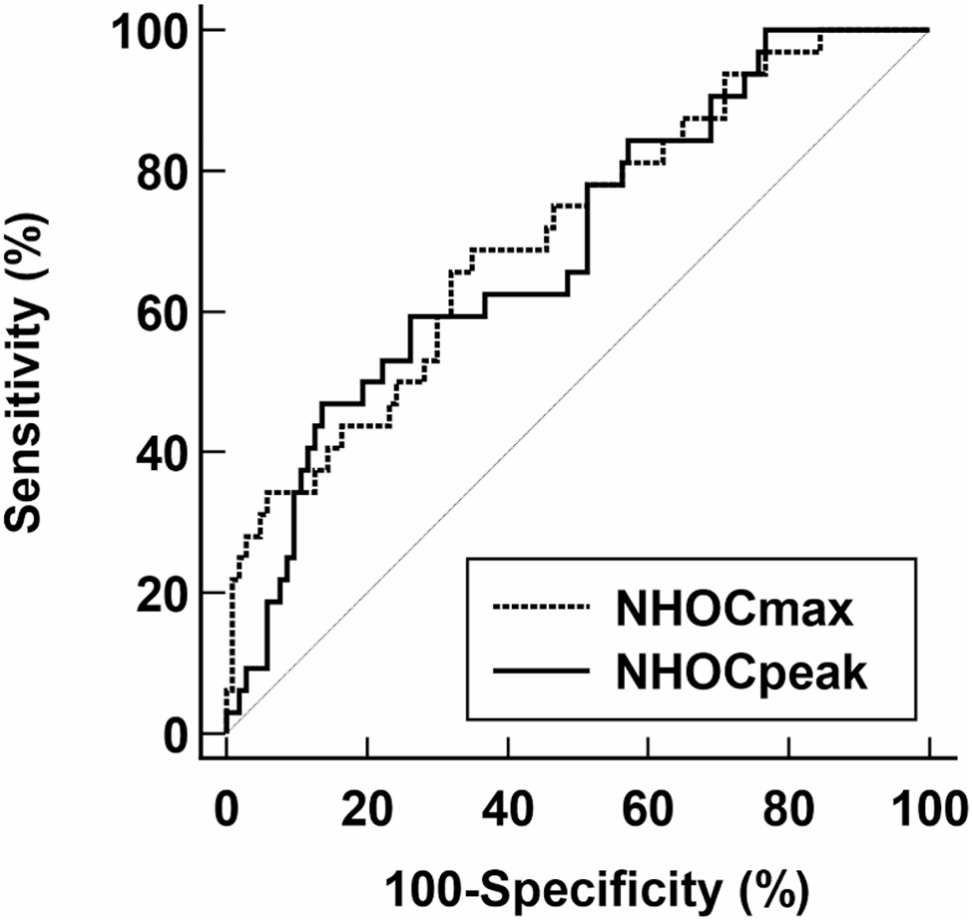
ROC curve analysis of NHOCmax and NHOCpeak for predicting pCR

In ROC curve analysis, NHOCmax demonstrated the highest value of AUC [0.710; 95% confidence interval (CI): 0.594–0.804] for predicting pCR among FDG PET/CT parameters, followed by NHOCpeak (AUC: 0.694; 95% CI: 0.584–0.792) (Table 3; Fig. 2). Both NHOCmax and NHOCpeak showed significantly higher values of AUC than SUVmax, SUVpeak, MTV, and TLG (*p* < 0.05). Both parameters demonstrated a high specificity (83.5% and 86.4%, respectively) for predicting pCR.

**Table 3:** ROC curve analysis of FDG PET/CT parameters for predicting pCR

Parameter	AUC (95% confidence interval)	Cut-off value	Sensitivity (%)	Specificity (%)
SUVmax	0.527 (0.408–0.634)	10.77	56.3	55.3
SUVpeak	0.518 (0.404–0.620)	6.98	65.6	44.7
MTV	0.597 (0.481–0.714)	4.93	43.7	73.8
TLG	0.558 (0.448–0.679)	56.22	59.4	54.4
NHOCmax	0.710 (0.594–0.804)	0.36	43.8	83.5
NHOCpeak	0.694 (0.584–0.792)	0.27	46.9	86.4
NHOPmax	0.639 (0.512–0.738)	0.31	56.2	72.8
NHOPpeak	0.578 (0.456–0.683)	0.22	71.9	43.9

**Fig. 3:**
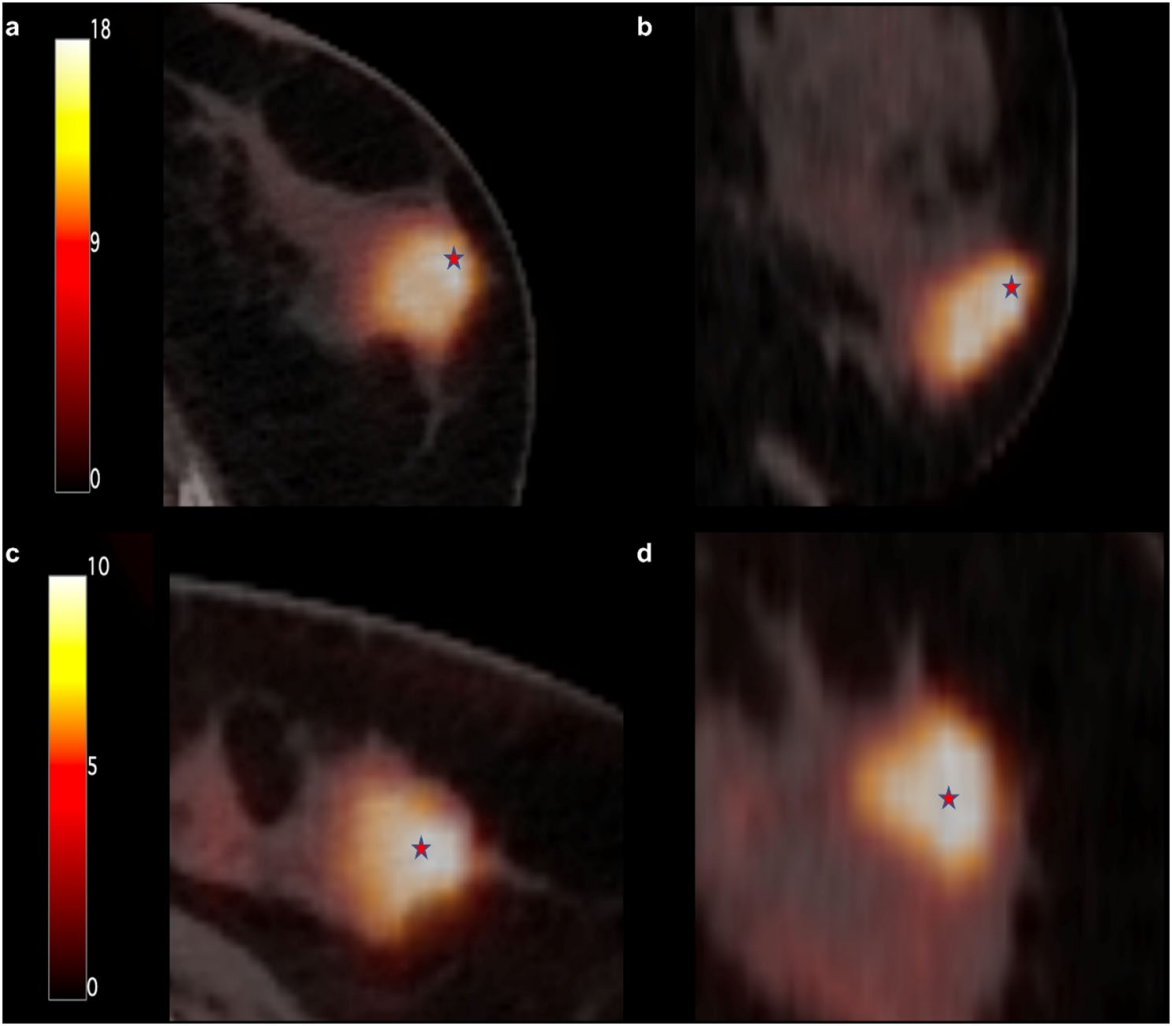
Transaxial (**A**) and coronal (**B**) FDG PET/CT images of a 52-year-old woman who was a non-responder to NAC with residual tumor cellularity of 50%. Her values of SUVmax, MTV, NHOCmax, and NHOCpeak were 21.63, 9.06, 0.78, and 0.75, respectively. Transaxial (**C**) and coronal (**D**) FDG PET/CT images of a 54-year-old woman who showed pCR to NAC. Her values of SUVmax, MTV, NHOCmax, and NHOCpeak were 10.83, 7.67, 0.27, and 0.23, respectively. Position of hot spot of FDG uptake is indicated by a red star

Representative cases of breast cancers with different NHOC parameters are shown in Fig. 3. In univariate logistic regression analysis, NHOCmax, NHOCpeak, and NHOPmax were significantly associated with pCR among PET/CT parameters (*p* < 0.05) (Table 4). These three parameters remained as independent predictors for pCR in multivariate logistic regression analysis after adjusting for age, T stage, N stage, and molecular subtypes (*p* < 0.05) (Table 4). Lower values of NHOCmax (*p* = 0.007) and NHOCpeak (*p* = 0.020) and higher values of NHOPmax (*p* = 0.021) were associated with higher pCR rates.

**Table 4:** Univariate and multivariate logistic regression analyses of FDG PET/CT parameters for predicting pCR

	Univariate analysis		Multivariate analysis	
Parameter	P-value	Odds ratio (95% confidence interval)	P-value	Odds ratio (95% confidence interval)
SUVmax	0.627	0.986 (0.931–1.044)		
SUVpeak	0.562	0.979 (0.913–1.051)		
MTV	0.116	0.976 (0.944–1.009)		
TLG	0.216	0.998 (0.994–1.001)		
NHOCmax	0.001	0.029 (0.004–0.219)	0.007	0.026 (0.003–0.214)
NHOCpeak	0.009	0.040 (0.005–0.308)	0.020	0.032 (0.004–0.282)
NHOPmax	0.021	35.375 (1.708–732.491)	0.021	40.010 (1.727–926.75)
NHOPpeak	0.135	8.882 (0.507–155.716)		

All p-values and odds ratios are for 1.00 increase of parameters

Each parameter in the multivariate analysis was separately incorporated in a multivariate model with adding age, T stage, N stage, and molecular subtypes as covariates

### Survival analysis

**Fig. 4:**
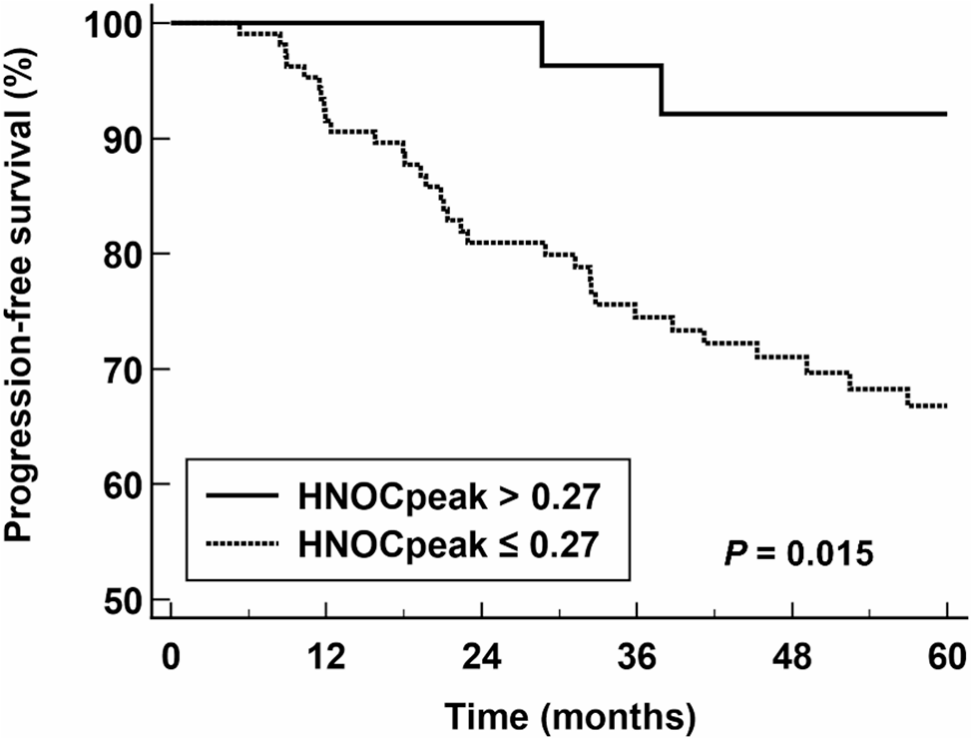
Kaplan–Meier curves of PFS according to NHOCpeak

In the univariate survival analysis for predicting PFS, it was found that SUVpeak, MTV, NHOCmax, and NHOCpeak were significantly associated with PFS (*p* < 0.05), whereas NHOP parameters failed to show statistical significance (*p* > 0.05) (Table 5). These four significant PET/CT parameters were included in the multivariate analysis with adjusting for age, T stage, N stage, and molecular subtypes. In the multivariate analysis, MTV (*p* = 0.020) and NHOCpeak (*p* = 0.026) were determined to be independent predictors for PFS (Table 5), revealing poor PFS in patients with increased values of MTV and NHOCpeak. In the Kaplan–Meier survival analysis using cut-off values from ROC curve analysis for predicting pCR, patients with high NHOCpeak (> 0.27) showed a significant worse PFS than those with low NHOCpeak (≤ 0.27) (5-year PFS rates: 66.8% vs. 92.1%; *p* = 0.015; Fig. 4).

**Table 5:** Univariate and multivariate survival analyses for PFS

	Univariate analysis		Multivariate analysis	
Parameter	P-value	Hazard ratio (95% confidence interval)	P-value	Hazard ratio (95% confidence interval)
SUVmax	0.058	1.936 (0.997–2.999)		
SUVpeak	0.047	1.916 (1.002–2.995)	0.310	1.803 (0.578–3.628)
MTV	0.004	1.012 (1.004–1.020)	0.020	1.010 (1.002–1.018)
TLG	0.058	1.001 (1.000–1.002)		
NHOCmax	0.041	3.311 (1.048–11.572)	0.255	2.112 (0.583–7.651)
NHOCpeak	0.019	4.023 (1.259–12.857)	0.026	4.455 (1.195–16.606)
NHOPmax	0.356	2.112 (0.378–25.387)		
NHOPpeak	0.461	2.401 (0.351–4.567)		

All p-values and hazard ratios are for 1.00 increase of parameters

Each parameter in the multivariate analysis was separately incorporated in a multivariate model with adding age, T stage, N stage, and molecular subtypes as covariates

## Discussion

During tumor growth, diverse genetic and epigenetic alterations inevitably occur, leading to intra-tumoral genetic heterogeneity with clonal evolution [18, 19]. Previous studies have found that more aggressive tumoral clonal population with higher cellular density and proliferation rates pushed toward the tumor edge as tumor grew because of compacted cell density and necrosis in the central area [13, 20]. Therefore, in the process of tumor progression, the areas containing cancer cells with high glucose metabolism would be switched from the center to the periphery of the tumor, showing displacement of the highest point of FDG uptake toward the peripheral side in tumors with aggressive features [13, 20]. Based on this finding, distances from the hot spot of radiotracer uptake to the tumor centroid and tumor perimeter have been proposed as novel geometrical parameters of FDG PET/CT in recent studies [13, 14]. To avoid dependence of tumor size, these parameters were divided by the radius of tumor lesion, which were defined as NHOC and NHOP, respectively [13]. In a recent study, NHOC and NHOP parameters were found to be little affected by voxel size and image postfiltering of PET images [14]. Furthermore, these parameters displayed only weak-to-moderate correlations (correlation coefficients of < 0.60) with conventional PET/CT parameters including SUVmax, SUVpeak, MTV, and TLG, suggesting that NHOC and NHOP parameters could be robust imaging parameters to bring additional information regarding tumor features [14]. Results of our study revealed significant relationships of NHOC and NHOP parameters with clinical T stage and triple negative cancer feature of breast cancer, supporting the concept that these geometrical parameters could reflect aggressiveness of cancer lesion. However, although both NHOC and NHOP parameters significantly correlated with clinical T stage and pathological response to NAC, since only NHOC parameters had significant associations with triple negative cancer type and residual tumor cellularity after NAC, NHOC parameters might be considered to be more suitable parameters for estimating tumor aggressiveness related with chemotherapy. On the other hand, histologic tumor grade and regional lymph node metastasis did not show significant associations with NHOC and NHOP parameters, which is consistent with a finding of a previous study of lung cancer patients [20]. Considering non-significant correlation results of NHOC and NHOP parameters with Ki-67 expression level, a well-known excellent marker of cell proliferation [21], these parameters might not have a direct relationship with proliferation rates of breast cancer cells. In a previous study employing a ring-type dedicated breast PET scanner, 24% of breast cancer lesions (18 out of 76 lesions) showed rim uptake pattern, which was correlated with tumor grade and triple negative cancer type [22]. Since breast cancer lesions with rim uptake pattern could be regarded as tumor lesions with increased NHOC parameters and decreased NHOP parameters, it can be suggested that distribution pattern of FDG uptake regarding spatial metabolic heterogeneity on breast cancer lesions reflects tumor aggressiveness [22].

In the present study, NHOCmax, NHOCpeak, and NHOPmax were independent predictors of pCR to NAC, with complete responders demonstrating lower NHOCmax and NHOCpeak values but higher NHOPmax values. Moreover, in ROC curve analysis, both NHOCmax and NHOCpeak showed significantly higher accuracy for predicting pCR than conventional PET/CT parameters, and these two parameters showed a high specificity of more than 80.0%. In previous studies, triple negative breast cancer and breast cancers with high histologic grade and Ki-67 expression level had better responses to NAC than other breast cancers [23, 24]. However, in other studies, breast cancers with aggressive features and necrosis showed poor responses to NAC [23, 25]. Although NHOC parameters had significant association with triple negative breast cancer in our study, our results suggested that NHOC parameters might reflect aggressive tumor features associated with a poor response to chemotherapy. Some previous studies have shown that conventional parameters of pretreatment PET/CT such as SUVmax and MTV were significant predictors for pCR to NAC in patients with breast cancer, whereas other studies have found only a poor predictive performance of conventional PET/CT parameters with an accuracy of 0.5 [8, 26–28]. To overcome these contradictory results, several recent studies have suggested that diverse textural features of primary tumor and metastatic lymph nodes regarding intra-tumoral metabolic heterogeneity could provide prognostic information regarding NAC response [7, 27, 29]. Results of our study indicated that NHOC parameters could also be significant PET/CT features for predicting pCR in breast cancer patients treated with NAC. Patients who showed high values of NHOCmax and NHOCpeak values could be considered as having a high risk for becoming non-responders to NAC with showing high residual tumor cellularity. Therefore, different management strategies might be needed according to values of NHOC parameters. Furthermore, since variances of PET/CT parameters after NAC have been shown to have superior predictive value than parameters of pretreatment PET/CT [6, 11, 28], dynamic changes of NHOC parameters during or after NAC might have further potential to predict NAC response and are worth investigating in future studies with breast cancer patients.

In the literature, three studies have investigated prognostic value of NHOC and NHOP parameters for predicting survival in patients with malignant diseases [13, 14, 20]. In studies on lung cancer patients, the normalized two-dimensional distance of SUVmax-to-tumor perimeter was significantly associated with short-term mortality in patients who received curative surgery, and both NHOCmax and NHOPmax were significant predictors for survival in those who received immunotherapy [14, 20]. Only a single study investigated prognostic significance of NHOCmax in 61 breast cancer patients, and found that patients with high NHOCmax showed worse disease-free survival and overall survival than those with low NHOCmax [13]. Similarly, in our study, both NHOCmax and NHOCpeak were significantly associated with PFS in univariate analysis, and NHOCpeak remained as a significant predictor for PFS in multivariate analysis after adjusting for age, T, stage, N stage, and molecular subtypes. Previous studies have demonstrated that textural features of breast cancer lesions regarding intra-tumoral metabolic heterogeneity significantly associated with survival outcomes as well as pCR to NAC [30, 31]. Furthermore, pCR and residual tumor cellularity after NAC, which showed significant correlations with NHOC parameters in the current study, are known to have significant associations with survival in patients with breast cancer [32]. Therefore, it is reasonable that NHOC parameters have significant prognostic value for predicting PFS. For breast cancer patients, NHOC parameters, rather than NHOP parameters, could be helpful in predicting both NAC response and risk of disease progression after treatment.

Our study has several limitations. First, this study was retrospectively performed with a relatively small number of patients, with only patients who had a sufficient tumor volume for calculating SUVpeak being enrolled. Hence, further validation with larger patient population is needed for generalizing results of this study. Second, because molecular subtypes and NAC regimens can also affect responses to NAC [32]; clinical significance of NHOC and NHOP parameters according to those factors should be further assessed. Lastly, although we excluded small tumor volume < 1 cm^3^, partial volume effect might have affected measurements of NHOC and NHOP parameters [14, 20].

## Conclusion

NHOCpeak measured from pretreatment FDG PET/CT images could predict pCR to NAC and PFS in patients with breast cancer. Increased NHOCpeak was associated with a lower pCR rate and a worse PFS. NHOC parameters could be promising novel geometrical parameters for predicting clinical outcomes in patients with breast cancer receiving NAC.

## Data Availability

The datasets used and/or analysed during the current study are available from the corresponding author on reasonable request.

## Abbreviations

AUC: Area under the receiver operating characteristics curve
CI: Confidence interval
FDG: F-18 fluorodeoxyglucose
HER2: Human epidermal growth factor receptor 2
MTV: Metabolic tumor volume
NAC: Neoadjuvant chemotherapy
NHOC: Normalized distance from the hot spot of radiotracer uptake to tumor centroid
NHOCmax: Normalized distance from maximum standardized uptake value to tumor centroid
NHOCpeak: Normalized distance from peak standardized uptake value to tumor centroid
NHOP: Normalized distance from the hot spot of radiotracer uptake to tumor perimeter
NHOPmax: Normalized distance from maximum standardized uptake value to tumor perimeter
NHOPpeak: Normalized distance from peak standardized uptake value to tumor perimeter
pCR: pathological complete response
PET/CT: Positron emission tomography/computed tomography
PFS: Progression-free survival
ROC: Receiver operating characteristic
SUVmax: Maximum standardized uptake value
SUVpeak: Peak standardized uptake value
TLG: Total lesion glycolysis
